# Cyperus (*Cyperus esculentus* L.): A Review of Its Compositions, Medical Efficacy, Antibacterial Activity and Allelopathic Potentials

**DOI:** 10.3390/plants11091127

**Published:** 2022-04-21

**Authors:** Shengai Zhang, Peizhi Li, Zunmiao Wei, Yan Cheng, Jiayao Liu, Yanmin Yang, Yuyan Wang, Zhongsheng Mu

**Affiliations:** 1 Institute of Economic Plants, Jilin Academy of Agricultural Sciences, Gongzhuling 136105, China; wodani999@163.com (S.Z.); miaomiao0825@126.com (Z.W.); chengyan199910@163.com (Y.C.); jiayaoliu@yeah.net (J.L.); wyy6677@126.com (Y.W.); 2 Binzhou Vocational College, Binzhou 256600, China; yanmin107@163.com; 3 Jia Sixie Agricultural College, Weifang University of Science and Technology, Weifang 262700, China; llppzz123@126.com

**Keywords:** Cyperus, *Cyperus esculentus* L., bioactive, medicinal efficacy, antibacterial activity, allelopathic potential, phytoinsecticide

## Abstract

Cyperus (*Cyperus esculentus* L.) is an edible perennial grass-like plant, which propagates exclusively with underground tubers. Its tubers are rich in starch (20–30%), fat (25–35%), sugar (10–20%), protein (10–15%) and dietary fiber (8–9%). In addition, the tubers also contain alkaloids, organic acids, vitamins (C and E), steroids, terpenoids and other active components. The contents of oleic acid and linoleic acid in Cyperus oil are very high, which have important medicinal value and health-promoting properties. Most of the extracts from the tubers, stems and leaves of Cyperus have allelopathic potential and antibacterial, antioxidant and insecticidal activities. In recent years, the planting area of Cyperus has increased significantly all over the world, especially in China and some other countries. This paper presents the current status of Cyperus and the recent trend in research in this area. Published reports on its nutritional contents, active ingredients, medicinal efficacy, antibacterial activity and allelopathic potential were also reviewed.

## 1. Introduction

**Figure 1:**
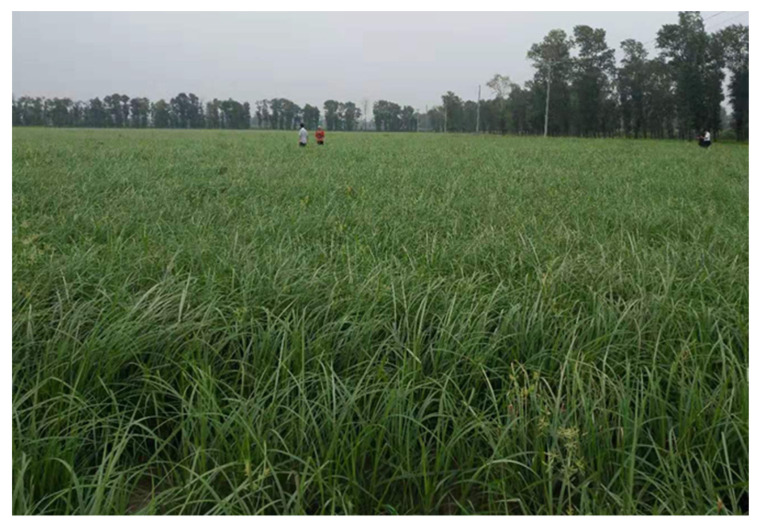
Cyperus (*Cyperus esculentus* L.) in growth stage.

**Figure 2:**
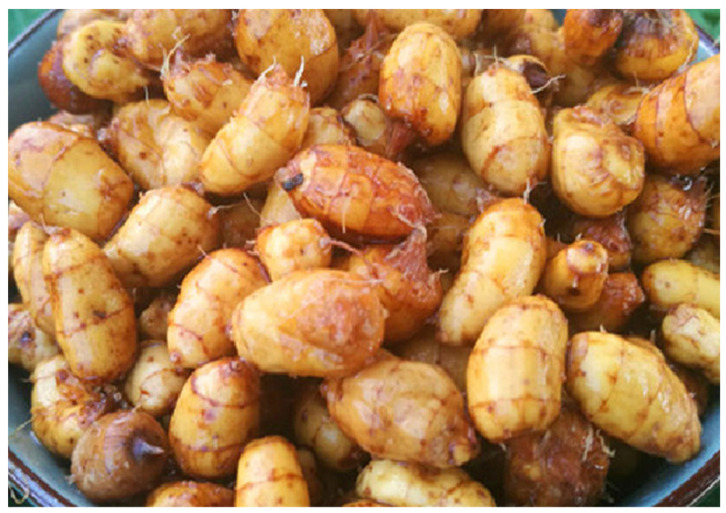
Cyperus (*Cyperus esculentus* L.) tubers at maturity.

Cyperus (*Cyperus esculentus* L.) is a perennial herb developed from rhizomes with sweet nut-like tubers, but cultivated species are often annual. It belongs to the genus Cyperus in the family Cyperaceae, and it propagates entirely with underground tubers in soil [1] (Figure 1 and Figure 2). Cyperus is also known by various other names, such as yellow nutsedge, tiger nut, chufa, iron water chestnut, underground chestnut, underground walnut, earth almond, ginseng fruit, ginseng bean, etc. [2,3]. Cyperus is rich in fat, protein, sugar, other nutrients and a variety of active substances. It is a new crop with high quality, high yield and high value [4]. The tuberous rhizomes of Cyperus have been used as food by hunter-gatherers and agricultural societies for millennia. 

Varieties and selections of the plant are widely grown in Southern Europe, North Africa and West Africa. Their popular uses as foods, beverage and medicine suggest that these edible tubers may have functional food potential. The ancient Egyptians first recognized the importance of this plant, planting and using it for culinary and medicinal purposes [5]. Later, the application of Cyperus became more and more extensive, from foods and medicine to its use in bioenergy [6]. In addition, there are a variety of active components in Cyperus, such as volatile oil, organic acids, alkaloids, phenols, terpenoids, anthraquinone, steroids, etc., which have antibacterial, antioxidant and antitumor activities. It was reported that the consumption of Cyperus could help prevent heart disease and thrombosis, improve blood circulation and lower the risk of colon cancer [7]. At the same time, a variety of active components in Cyperus have strong allelopathic effects, which have a great impact on intercropping, interplanting and crop rotation. However, due to its grass-like appearance and the poor research on Cyperus, for a long time in the past, some countries had always treated it as a weed, so it had not been fully utilized and investigated [8]. In recent years, it has been widely cultivated in Spain, Africa, Australia, South and North America. The annual value of Cyperus production in Spain has risen to 3.3 million euros, and its production area has also increased significantly in China, Italy, Bulgaria, etc. At present, there is still a lack of understanding in the active components, antibacterial activity and allelopathic potential of Cyperus, which affects the development, utilization and cultivation of Cyperus. Therefore, this paper reviews the chemical composition, medical efficacy and allelopathic potential of Cyperus to provide the references for the in-depth research, further development and utilization of Cyperus.

## 2. Compositions

Cyperus is a new, high-quality oil crop. It is rich in oil, starch and sugar, and it also contains protein, dietary fiber, vitamin C, vitamin E and a variety of minerals (e.g., phosphorus, potassium, magnesium, sodium, calcium, etc.) (Table 1). From Table 1, (i) the content of lipid in Cyperus is higher than that of soybean. It can be used as a new oil crop and has great development potential; (ii) the content of starch in Cyperus is significantly higher than that of common oil crops, up to 23.2–29.9%; (iii) the contents of starch and carbohydrate in Cyperus are significantly higher than that of common oil crops, which indicates that Cyperus is a food of raw materials with high calories. 

In addition, Cyperus also contains a variety of active substances (e.g., alkaloid, saponin, phytosterol, flavone, terpenoid, tannin, etc.) [25]. These active substances are important pharmaceutical components. Most flavonoids have strong biological activities, such as an antioxidant effect, and can be used in pharmacological research (Table 2).

Due to the differences in climate, soil and cultivation conditions, the bioactive content in Cyperus varies. It was reported that, under normal conditions, the crude fat content in Cyperus tuber (dry tuber) is 25–35%, crude protein 10–15%, starch 20–30%, sugar 10–20%, vitamin E 0.8–1.4%, and the flavonoids in stems and leaves could reach 13.2 mg/g [11,27]. Chen et al. found that the contents of fat, starch, sugar, protein and water in the tubers of Cyperus were 26.5%, 23.2%, 23.4%, 8.0% and 7.0%, respectively, and other active components (e.g., organic acids, terpenoids, steroids, etc.) were also detected [29].

Liu showed that the basic components of Cyperus were 26.5% fat, 23.2% starch, 23.4% sugar, 8.0% protein and 7.0% water. At the same time, she also detected organic acids, vitamins, steroids and terpenes with health care effects, in which the content of vitamin E was 0.15% and the content of sterols was 0.53% [30]. The test results of Chen et al. and Liu are very similar. It can be concluded that the contents of fat, starch and sugar in Cyperus are indeed very high. In addition, Yan et al. detected the main components in the stems and leaves of Cyperus. The results showed that the stems and leaves of Cyperus contained 2.53% crude fat, 7.08% crude protein, 47.1% crude fiber, 10.5% ash and 7.75% sugar (in glucose) [26]. It also contained peptides, phenols, organic acids and alkaloids. These active ingredients play an important role in the fields of medicine and ecology [31]. Yan et al. conducted a qualitative analysis on the active components of Cyperus. The results showed that Cyperus contained organic acids, alkaloids, amino acids, peptides, polysaccharides, volatile oils, phenols, terpenes, anthraquinones, steroids and other active components [26]. Chukwuma et al. determined the phytochemical composition of Cyperus tubers. The results showed the presence of resins, alkaloids, cyanogenic glycosides, tannins, sterols and saponins were observed in the raw tubers, while only sterols, resins and alkaloids were observed in the roasted tuber. Meanwhile, analysis of the composition yielded saponins (0.88 ± 0.02 mg/100 g), tannins (9.50 ± 0.46 mg/100 g), oxalates (0.25 ± 0.65 mg/100 g), phytate (1.97 ± 0.81 mg/100 g) and cyanogenic glycosides (1.80 ± 0.69 mg/100 g) [7].

Cyperus (*Cyperus esculentus* L.) oil is golden in color, clear and transparent, and has a strong aroma. It is a rare raw material resource in the field of food, health products and cosmetics [32] (Table 3). From Table 3, the oleic acid content of Cyperus oil is significantly higher than that of common vegetable oils, and it does not contain erucic acid. Therefore, it is a healthy edible oil. 

Cyperus oil contains 64–73.3% oleic acid and 11–15.5% linoleic acid, which is similar to olive oil (55–83% oleic acid and 3.5–21% linoleic acid) and is significantly higher than the oil contents in soybean, peanut and rapeseed oils [2,37]. It was found that Cyperus oil also contains active components such as rapeseed sterol, stigmasterol, 7-cholesterol, B-sitosterol, 24 methylene-Δ7-cholesterol, 5-oatsterol and cycloatroxanol. These plant components are important raw materials for the pharmaceutical industry [38,39].

## 3. Medicinal Research

### 3.1. Medicinal Efficacy

It is reported that the tuber of Cyperus (*Cyperus esculentus* L.) possesses an irregular fruit shape, rough surface, pungent smell, bitter taste and cool property. It has important medicinal value [25]. In India, Cyperus tuber is the oldest medicinal material, which can be used to treat critical and serious diseases (e.g., chronic gastritis, lymphatic tuberculosis, burns and scalds, coronary heart disease, acute cholecystitis, acute intracerebral hemorrhage, etc.) [40]. According to the *Xinhua outline of Materia Medica*, the tuber of Cyperus contains a kind of spicy, sweet and warm nature. It has the functions of soothing the liver, aerating and strengthening the spleen and stomach, and it can also prevent many diseases (e.g., obesity, diabetes, gastrointestinal diseases, etc.). The active ingredients of Cyperus can be used to treat chest tightness, dyspepsia, food stasis, female menstrual disorders, and so on. In Indian medical records, Cyperus has similar medicinal effects, too [41,42]. Another study showed that Cyperus contains an active component, anthraquinone (C14H8O2), which has the effects of anti-tumor, anti-oxidation and sterilization [43]. Achoribo et al. found, in their experiment, that the oleic acid contained in Cyperus oil can inhibit the overexpression of “Her-2/neu”, thereby promoting the apoptosis of breast cancer cells by using “Her-2/neu” oncogene amplification [44].

Cyperus also has the effects of protecting nerves, the stomach and kidneys, strengthening yang and treating anemia. Wiebe et al. undertook a series of studies in order to indicate the central nervous system activity of the extract (Cyperol) from Cyperus (*Cyperus esculentus* L.). The results showed that Cyperol could induce changes in brain electrical activity (EEG), demonstrating that active ingredients from the extract were absorbed and were bioavailable. In summary, the results of the study indicate calming effects without sedation. The extract (Cyperol) does indeed have functional food potential and can be used to maintain a calm state of mind while dealing with cognitively demanding works [45]. Jing et al. studied the intervention effect of orientin in Cyperus on nerve injury in rats. The results showed that the orientin could significantly improve the neurological function of rats [46]. Elshamy et al. studied the effects of different concentration extracts from Cyperus on gastric ulcers induced by ethanol in rats. The results showed that the protective effect of the extract on gastric epithelial cells increased with the increase in concentration [47]. Imo Chinedu et al. examined the effects of ethanolic extracts from Cyperus (*Cyperus esculentus* L.) on selected indices of kidney function in male albino rats. The results showed the extracts had no apparent toxic effect on the kidney function of the experimental rats. The results also showed the extracts may help to reduce the retention of urea, thereby strengthening kidney functions. This plant material is therefore encouraged for use in general nutrition [48]. Nto et al. studied the intervention effect of Cyperus extracts on testicular dysfunction in Wistar rats. The results showed that Cyperus extracts significantly increased the number and volume of the epithelial cells. This finding indicates that Cyperus extracts can improve the vitality of testis in Wistar rats [49]. Jagpal studied the effects of Cyperus oils on reducing cell sickling in sickle cells anemia in vitro. The results showed that the Cyperus oil treatments resulted in an increase in the antioxidant presence of sickle cell samples when tested in vitro, as well as a morphological decrease in sickle cells and increase in spherical erythrocytes, thereby alleviating the symptoms of anemia [50]. In addition, Cyperus tubers (*Cyperus esculentus* L.) contain various compounds with intestinal health-promoting properties. Moral Anter David et al. studied the effect of Cyperus on *S. enteritidis* agglutination, oxidative stress and lactobacillus plantarum growth. The results revealed that, compared to controls, Cyperus partially restored “TER” in *S. enteritidis* infected cultures and promoted the growth of lactobacillus plantarum [51].

### 3.2. Health-Promoting Properties

Cyperus (*Cyperus esculentus* L.) is rich in fat, and the edible oil (Cyperus oil) can be extracted from it. Cyperus oil has a mellow taste and stable chemical properties, and it is clear and transparent, not easy to corrupt and very easy to be absorbed and utilized by the human body. It is a very high-quality edible oil [52]. The main components of Cyperus oil are unsaturated fatty acids (oleic acid and linoleic acid). Linoleic acid is an essential fatty acid for the human body. It is an important substance for the synthesis of prostaglandins. It can regulate physiological functions of the human body, promote the growth and development of the human body, promote the catabolism of cholesterol in the body, and it can prevent cardiovascular diseases. Cyperus oil is also rich in minerals, but the content of sodium is very low and does not contain cholesterol, which is very close to olive oil, hazelnut oil and avocado oil [53]. It is also reported that the content of sterols in Cyperus oil is about 1000 μg·g^−1^, which is significantly higher than that in olive oil (about 100 μg·g^−1^). These sterols in Cyperus oil could reduce the absorption of cholesterol and reduce the risk of cardiovascular disease [30]. Bamishaiye et al. studied the effects of Cyperus oil and soybean oil on tissue metabolism in rats. The results showed that, compared with feeding soybean oil, the treatments of feeding Cyperus oil significantly reduced the content of total cholesterol in rats [54]. Moon et al. studied the effect of Cyperus oil meal on lipid metabolism in mice. The results showed that Cyperus oil meal significantly reduced the increase of adipocyte volume, adipose tissue weight and body weight caused by high-fat diet, and it significantly reduced the contents of total cholesterol and triglyceride in serum and triglyceride in liver. The results showed that Cyperus is beneficial in preventing hyperlipidemia caused by diet [55]. Salem M L et al. studied the effects of Cyperus tubers on mice with atherosclerotic disease. The results showed that feeding Cyperus tubers to mice significantly alleviated the symptoms of atherosclerosis in mice [56]. According to research by Oluwajuyitan and others, Cyperus is a food that lowers the glycemic index, and it is suitable for diabetics [57]. Another study showed that Cyperus is a pollution-free and nutritious food raw material with the function of protecting the liver [58]. Onuoha et al. studied the protective effect of Cyperus milk on liver injury induced by acetaminophen (APAP). The results showed that Cyperus milk could significantly improve liver injury induced by acetaminophen (APAP) [59]. Hassanein et al. studied the protective effect of Cyperus essential oil on the hepatotoxicity induced by paracetamol. The results showed that the concentration of the essential oil at 12.5 μg/mL has a protective effect on the hepatotoxicity induced by paracetamol [60].

### 3.3. Antibacterial Activity

It is reported that Cyperus (*Cyperus esculentus* L.) contains a variety of active components, and most of its extracts have antibacterial effect [41]. Prakash et al. prepared different extracts of Cyperus, such as acetone, 50% ethanol, chloroform and petroleum ether to evaluate for their antibacterial activity against several human pathogens (*Escherichia coli*, *Staphylococcus aureus*, *Salmonella* sp, *Klebsiella pneumoniae*, *Proteus vulgaris*, *Pseudomonas aeruginosa* and *Citrobacter freundii*) by using the disc diffusion method. The acetone extract showed the highest inhibitory activity against *S. aureus*, *ILpneumoniae* and *P. vulgaris*. Furthermore, 50% ethanolic extract showed the maximum activity against *Escherichia coli*, *S. aureus* and *Salmonella* sp. Chloroform extract maximally inhibited the growth of *S. aureus*, whereas petroleum ether extract showed positive results against *Salmonella* sp., respectively. All extracts were sensitive to *C. freundii* [61]. Abdel et al. evaluated the antibacterial activity of Cyperus (*Cyperus esculentus* L.) oil via cup plate agar diffusion assay against six standard human pathogens (Gram positive: *Staphylococcus aureus* and *Bacillus subtilis*; Gram negative: *Escherichia coli*, *Pseudomonasa aeruginosa*, *the fungi Candida albicans* and *Aspergillus niger*). The oil showed different antimicrobial responses against testing organisms. It showed significant activity against the fungus *Candida albicans* and partial activity against *Staphylococcus aureus* [62]. Salem et al. studied the effect of Cyperus tubers on the reduction in atherosclerotic lesions in mice. The results indicated that the ingredients of Cyperus tubers exhibit anti-inflammatory properties upon inflammation and immunostimulatory effects in immunocompetent hosts [56].

### 3.4. Antioxidant Activity

Some studies showed that Cyperus (*Cyperus esculentus* L.) contains secondary metabolites of flavonoids, which showed antioxidant activity and an anticoagulant effect [63]. Jing et al. studied the antioxidant activity of the extracts from Cyperus. The results showed that the activities of superoxide dismutase (SOD) in liver tissue and serum of mice were significantly increased by different concentrations (high, medium and low) [64]. Willis et al. studied the antioxidant activities of water and methanol extracts from Cyperus. The results showed that both extracts had scavenging effects on free radicals [65]. Badejo et al. mixed Cyperus milk, ginger juice and water (7:1:2) to make a Cyperus mixed beverage, and they studied its effect of scavenging free radical. The results showed that the scavenging rate of free radical (DPPH) was 32.53% [66]. Another study found that the germinated tubers of Cyperus could further improve antioxidant activity [67,68]. Siqun Jing et al. evaluated the antioxidant activity and antibacterial property of the flavonoids from Cyperus (*Cyperus esculentus* L.) leaves. In all the assays, Cyperus leaves flavonoids had pronounced antioxidant activity in vivo that could significantly elevate the content of superoxide dismutase (SOD). Cyperus leaves flavonoids inhibited the growth of both Gram-positive and Gram-negative bacteria. The results indicate that the flavonoids from Cyperus leaves can be taken as a natural antioxidant and bacteriostatic substance in the food and pharmaceutical industry [69].

## 4. Allelopathic Potential

### 4.1. Impacts on Crops

It is reported that Cyperus has a strong allelopathic effect (inhibition) on many crops [70]. Dirk et al. studied the allelopathic effect of extracts and plant residues of Cyperus (*Cyperus esculentus* L.) on the growth of corn (*Zea mays* L.) and soybean (*Glycine max* L.) and found that tuber residues reduced the dry weight of corn and soybean more than foliage residues. As the concentration increased, the growth decreased, affecting soybean more than corn. Soybean growth was significantly reduced by the addition of tuber extracts. Growth inhibition was greatest when the tuber residue was in contact with the corn or soybean seeds [71]. This shows that extracts and residues of Cyperus have an allelopathic effect on corn and soybean. Reinhardt et al. studied the allelopathic effects of Cyperus (*Cyperus esculentus* L.) tubers on ectomycorrhiza (*Boletus*), lettuce (*Lactuca sativa*) and corn (*Zea mays*), and they further evaluated the allelopathic potential of Cyperus. The results showed that the aqueous extracts from Cyperus tubers significantly reduced the colony diameter of the ectomycorrhiza (*Boletus*) on agar medium. At 2% concentration, the aqueous extracts inhibited the germination of lettuce (*Lactuca sativa*) seeds significantly. The emergence of corn (*Zea mays* L.) was retarded in soil where Cyperus tubers were planted 28 days before planting the crop, irrespective of whether the Cyperus tubers continued to grow or were physically removed at the time the corn was sown. This shows that the root exudates or residues of Cyperus have an inhibitory effect on corn seed germination [72]. Later, H.P. Oamen et al. studied the allelopathic effects of aqueous extracts of *Cyperus esculentus* L. on the germination and seedling growth of *Cajanus cajan* L., *Phaseolus vulgaris* (brown and white varieties) and *Sphenotylis stenocarpa*. The results showed that the extracts have significant positive allelopathic effects on plumule and radicle lengths at *p* < 0.05. The number of root hairs for *C. cajan* was reduced at higher concentrations [73]. Papadi Asimina et al. studied the allelopathic potential of Cyperus (*Cyperus esculentus* L.) on the germination of the seeds of six commonly grown crops (*Zea mays* L., *Citrullus lanatus Thunb*, *Abelmoschus esculentus* (L), *Vigna unguiculata* (L), *Glycine max* (L) and *Arachis hypogaea* (L.). The results showed that the aqueous extracts from Cyperus reduced the germination counts of the seeds by 10 to 100%. Cyperus shoot extracts were the most phytotoxic. In addition, the decomposing mulches showed varied but less inhibitory effects on the seeds with a trend toward increasing the inhibitory effect with an increasing mulch level and decreasing seed size [70]. Singh C.M. et al. studied the allelopathic effects of Cyperus (*Cyperus esculentus* L.) on the seed germination and seeding growth of wheat. The results showed that the extracts from Cyperus had inhibitory effects on the germination of wheat seeds. The time taken for germination prolonged; root, shoot and leaf developments were seriously inhibited. The findings of this study clearly indicate that before using the Cyperus for manuring in wheat fields, it would be safe to carry out a detailed field experiment to ascertain the allelopathic effects [74]. JJ Sani et al. evaluated the allelopathic effects of extracts from the aerial and underground organs of Cyperus (*Cyperus esculentus* L.) with three levels of extract (5, 10 and 20 percent) on rapeseed germination and growth. The results showed that the extract of Cyperus reduced rapeseed germination and growth. With increasing concentration of extracts, germination rate, germination percent, vigor index, root and shoot length were significantly reduced [75]. M.G. Patterson et al. conducted a series of experiments, from 1976 to 978, to determine the competitive relationship of Cyperus (*Cyperus esculentus* L.) with cotton. In the experiments, Cyperus was left undisturbed or removed from plots to give periods of competition of 2, 4, 6, 8, 10 and 25 weeks. The results showed that the seed cotton yield was reduced in 2 out of the 3 years in a full season (25 weeks) of competition but was unaffected by shorter periods of competition in all years. The main stem diameter of cotton was also reduced 2 out of the 3 years when competing the full season, while the main stem height was reduced in only 1 out of the 3 years in a full season competition [76].

### 4.2. Phytoinsecticide

Avicor et al. investigated the larvicidal potential of crude aqueous extracts from two Cyperus (*Cyperus esculentus* L.) varieties (black and yellow) on the mosquitoes *Aedes aegypti* (L.) and Culex *quinquefasciatus* (Say). Differential larvicidal responses were observed in the test mosquitoes, and extracts of black dried Cyperus were more larvicidal than yellow dried Cyperus. The results indicated the potential of Cyperus, particularly black dried Cyperus, as a resource of mosquito insecticide [77]. Based on the research results in this area, Cyperus (*Cyperus esculentus* L.) has great potential in developing phytoinsecticide and reducing people’s dependence on synthetic insecticides.

## 5. Future Areas of Research

The following studies need be conducted in the future: (i) Preliminary indications suggest that the extract (Cyperol) from Cyperus (*Cyperus esculentus* L.) may have anxiolytic potential [45], which should be explored in clinical studies. (ii) The extraction and identification of allelochemicals from Cyperus. (iii) The allelopathic effects of extracts and decomposed substances from Cyperus on main crops (e.g., soybeans, corn, wheat, etc.) and its autotoxicity. (iv) The allelopathic effects of extracts and substances from decomposed Cyperus on soil microflora and activity. (v) The development of new allelopathic varieties of Cyperus. (vi) The larvicidal potential and identification of the extracts from Cyperus. Furthermore, (vii) studies on the medicinal efficacy and antibacterial potential of Cyperus should also be conducted in the future.

## 6. Conclusions

Some studies have been carried out on the chemical components, active substances and allelopathy of Cyperus (*Cyperus esculentus* L.), and it has been found that it has medicinal efficacy and antibacterial potential, but these studies are not detailed and in-depth enough. Therefore, it is necessary to study and evaluate its active components and antibacterial and insecticidal potential in more detail, in order to provide a scientific basis for the further development and utilization of Cyperus. In addition, Cyperus has strong allelopathy to many crops, and it inhibits the growth of adjacent crops. At the same time, Cyperus (*Cyperus esculentus* L.) has great potential in developing phytoinsecticide. Research on the allelopathy of Cyperus has a bright future in reducing our reliance on synthetic pesticides and promoting the sustainable development of Cyperus cropping.

## Figures and Tables

**Table 1:** Main composition and content of cultivated Cyperus (*Cyperus esculentus* L.) tubers compared to these of key oil crops.

Product	Cyperus (%)	Peanut (%)	Soybean (%)	Rape (%)	Sunflower (%)	Sesame (%)
Scientific name	*Cyperus esculentus*	*Arachis hypogaea*	*Glycine max*	*Brassica napus*	*Helianthus annuus*	*Sesamum indicum*
Starch	23.2–29.9	4.00	2.00–6.53	0.80–1.18	12.0–18.0	–
Lipid	20.1–34.5	39.2–49.8	16.0–22.0	37.5–46.3	49.9–51.2	44.9–57.9
Protein	5.04–8.00	20.3–26.7	35.0–44.6	23.1–32.4	15.4–23.9	18.4–30.4
Crude Fiber	8.06–8.91	3.30–8.50	5.23–15.5	5.7–9.6	4.90–6.10	7.45–12.6
Carbohydrate	43.3	7.48–22.1	22.0–30.0	15.9–24.8	13.0–16.7	14.4–31.5
Total sugar	15.4–23.4	3.54–6.53	5.06–7.70	8.00–12.9	16.0–23.0	8.44–12.3
Ash	1.60–2.19	2.02–2.27	4.35–4.60	4.10–5.30	3.20–5.40	4.77–6.18
Moisture	6.13–7.27	6.20–7.30	10.2–11.1	8.05	3.00–3.82	3.06–6.15
References	[9,10,11,12]	[13,14,15]	[16,17,18]	[19,20]	[14,21,22]	[23,24]

Note: “–” indicates no report.

**Table 2:** Main active substances of cultivated Cyperus (*Cyperus esculentus* L.) tubers.

Ingredient	Concentration	Reference
Resins	+++	[7,26]
Cyanogenic glycosides	+	[7,26]
Alkaloids	+++	[7]
Glycosides	0	
Flavonoids	+++	[27,28]
Saponins	+	[7]
Tannins	+	[7]
Sterols	+++	[7]
Cardiac glycosides	0	
β-sitosterol	+++	[26]
δ^5^-avenasterol	+	[26]
Vitamin E	+	[26]

Note: ‘+++’ indicates very high concentration, ‘++’ indicates medium concentration, ‘+’ indicates trace concentration and ‘0′ indicates uncertainty.

**Table 3:** Main fatty acid composition and content of cultivated Cyperus (*Cyperus esculentus* L.) oils compared to those of common vegetable oils (Crude oil).

Product	Cyperus (%)	Peanut (%)	Soybean (%)	Rape (%)	Sunflower (%)	Sesame (%)
Scientific name	*Cyperus esculentus*	*Arachis hypogaea*	*Glycine max*	*Brassica napus*	*Helianthus annuus*	*Sesamum indicum*
Palmitic acid (C16:0)	11.7–14.2	9.7–13.0	6.0–9.3	5.0	5.5	8.2
Stearic acid (C18:0)	1.1–4.9	2.7–5.0	3.0–5.5	3.6	2.9	1.9
Oleic acid (C18:1)	67.7–76.6	41.0–47.1	24.8–36.0	14.0–19.0	15.0–28.9	35.0–49.4
Linoleic acid (C18:2)	8.8–11.5	37.6	52.0–65.0	12.0–25.4	62.0–70.0	37.7–49.1
Linolenic acid (C18:3)	0.2–1.9	–	2.0–6.8	7.3–10.0	0.3	0.4
Arachidic acid (C20:0)	0.4–6.1	1.8–8.0	0.1–0.4	0.4–1.0	0.2	0.4–1.2
Erucic acid	–	–	0.23	31.0-55.0	–	–
References	[25,33,34]	[35,36]	[35,36]	[35,36]	[35,36]	[35,36]

Note: “–” indicates no report.

## Data Availability

Not applicable.
